# The Precapillary Segment of the Blood-Brain Barrier and Its Relation to Perivascular Drainage in Alzheimer's Disease and Small Vessel Disease.

**DOI:** 10.1100/tsw.2009.72

**Published:** 2009-07-04

**Authors:** Dietmar R. Thal

**Affiliations:** Institute of Pathology, Laboratory of Neuropathology, University of Ulm, Germany

**Keywords:** blood-brain barrier, perivascular space, apolipoprotein E, small vessel disease, Alzheimer's disease

## Abstract

The blood-brain barrier (BBB) is the border between the brain tissue and the blood and consists of a pre- and post-capillary and a capillary segment. At capillaries protein transport is possible via receptor-mediated endocytosis through endothelial cells. At the arteries and veins the BBB is thicker and there is, under physiological conditions, no direct transport from the brain tissue to the blood or vice versa. Here, extracellular fluid is drained into the perivascular space, which is a fluid-filled space between the border of the brain tissue, the glia limitans, and that of the vessel wall, the adventitia as well as along basement membranes within the vessel wall. In the event of degenerative changes in the arterial vessel wall, known as small vessel disease (SVD), leakage of plasma proteins into the vessel wall and into the perivascular space occurs. Thus, the pre-capillary segment of the BBB is altered and drainage of extracellular fluid from the brain tissue competes with the leaking plasma for perivascular drainage. Since the amyloid ß-protein (Aß) is subject of this perivascular drainage and accumulates in the brains of Alzheimer's disease (AD) patients, an alteration of this drainage system in the course of SVD may support the accumulation of Aß within the brain and, in so doing, the development of AD.

## INTRODUCTION

Perivascular drainage of the extracellular fluid of the brain is one of the clearance mechanisms for proteins in the brain[1]. Other clearance mechanisms are cellular uptake with subsequent lysosomal degradation or transport through the capillary segment of the blood-brain barrier (BBB) as described, for example, for the amyloid β-protein (Aβ)[2,3,4,5,6,7,8,9].

The morphological correlatives for perivascular drainage of interstitial fluid are the perivascular spaces[1] and the basement membranes within the vessel walls[10,11]. Proteins that are produced in the brain are cleared into the perivascular space[12]. The perivascular fluid is further drained either into the cerebrospinal fluid (CSF) or into cervical lymph nodes[1]. Thus, this drainage mechanism is mainly located in the noncapillary segments of the BBB and seems to be involved in the pathogenesis of brain diseases, such as Alzheimer's disease (AD) or neuroinflammatory disorders[1,2,11]. Under normal conditions, pericytes control the postendothelial permeability of the BBB to the perivascular space, especially in the pre- and postcapillary segment[2]. The glia limitans represents a barrier between the brain parenchyma and the perivascular space[2].

This review is focused on the precapillary segment of the BBB and describes its anatomy, function in protein clearance, and its relation to AD and small vessel disease (SVD). A possible pathogenetic role of alterations in the precapillary segment of the BBB and the perivascular drainage for the development of AD is discussed.

## ANATOMY OF THE BBB AND ITS RELATION TO PERIVASCULAR DRAINAGE

The morphological correlatives of perivascular drainage channels are the perivascular spaces in the pre- and postcapillary segments of the BBB, and the basement membranes in all segments of the BBB (Fig. 1)[11,12,13]. The perivascular space is usually bordered by the glia limitans of the brain and adventitial pericytes[2] (Fig. 1). In larger vessels of the basal ganglia, the glia limitans and the adventitia are covered by a thin layer of the pia mater in contrast to smaller brain vessels[14]. In comparison to the BBB capillary segment consisting of the endothelium, the basement membrane with pericytes, a minimal perivascular space, and the glia limitans, the vessels of the pre- and postcapillary segment have a lamina media and a significant adventitia in the vessel wall and larger perivascular spaces[2] (Fig. 1). At the capillary segment, the endothelium has the major barrier function and regulates fluid and protein transport[2,15,16,17,18]. The blood pressure in the capillaries is very low and the endothelial cell layer is usually not altered by the blood pressure. In the precapillary segment, on the other hand, the blood pressure is relatively high because these vessels are arteries or arterioles (Fig. 1). Therefore, a pressure-related, mechanical alteration of the endothelial cell layer is possible, leading to the leakage of blood plasma into the vessel wall as similarly described as the first step in the pathogenesis of atherosclerosis[19]. In so doing, the lamina media and adventitia, the perivascular space, as well as the glia limitans, become more important for the efficiency of BBB function in the precapillary segment. The postcapillary segment can be altered in the course of inflammatory disorders, e.g., perivenous encephalomyelitis (= acute disseminated encephalomyelitis).

## PERIVASCULAR CLEARANCE OF Aβ AND APOE IN THE NORMAL BRAIN

Extracellular fluid in the brain is drained from the brain parenchyma either into the CSF or into perivascular channels, finally ending at the cervical lymph nodes[1]. Thus, proteins in the extracellular space are also subject to perivascular clearance, including Aβ (a protein that accumulates in the brains of AD patients in the form of extracellular senile plaques) and apolipoprotein E (apoE, a protein that is involved in lipid and Aβ transport; the APOE ε4 allele is a risk allele for AD). For Aβ as well as for apoE, it could be shown that the proteins produced by astrocytes (apoE) or neurons (Aβ) occur in the perivascular space of transgenic mice expressing a human-specific form of apoE in astrocytes[12], or producing Aβ due to an overexpression of neuronal amyloid precursor protein (APP)[20]. Under normal conditions, significant amounts of apoE can be detected in the perivascular space, whereas only low levels of Aβ were detectable in wild-type mice as well as in nondemented human autopsy cases[12,21]. Moreover apoE receptors, such as the $\alpha_2$ macroglobulin receptor/low-density lipoprotein receptor-related protein (LRP1), are expressed in glial cells and along the BBB, and take up apoE-linked Aβ[3,5,22,23,24,25,26]. Therefore, it is tempting to speculate that apoE is physiologically involved in transport and/or clearance mechanisms for, for example, lipids and Aβ through the perivascular space.

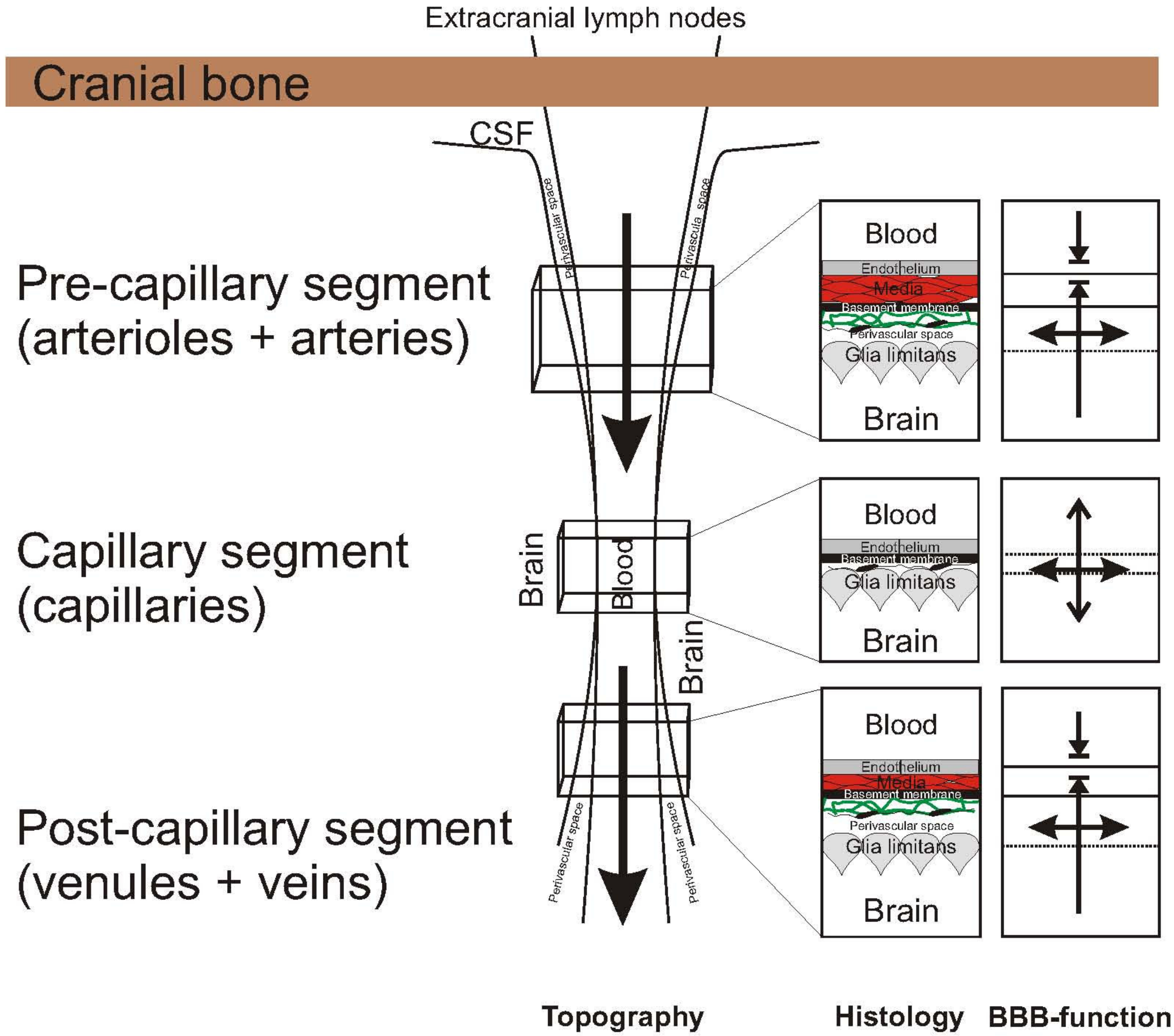

**FIGURE 1**. Schematic representation of the three segments of the BBB. *Topographically*, the precapillary segment of the BBB is associated with the intracerebral arteries and arterioles, whereas the postcapillary segment is associated with intracerebral venules and veins. The capillary BBB segment represents all capillaries of the brain parenchyma. Arteries and veins enter the brain from the extracranial site. As demonstrated in the schematic representation of the *histology*, the perivascular space is significant in the pre- and postcapillary segment, whereas it is negligible in the capillary one. *Functionally*, the endothelium of the pre- and postcapillary segments does not allow significant transport of fluid or proteins from the blood into the perivascular space and the brain parenchyma, or vice versa. Even the high arterial blood pressure in the precapillary segment does not lead to plasma leakage under normal conditions. However, extracellular fluid and proteins can be cleared into the perivascular space and into vascular basement membranes from where it is drained into the CSF or to the extracranial cervical lymph nodes[1,10].

## PERIVASCULAR CLEARANCE OF Aβ AND APOE IN THE AGED AND THE AD BRAIN, AND ITS RELATION TO CEREBRAL SVD

Since apoE and Aβ are cleared through the perivascular space and possibly through the vessel walls along the basement membranes, it should be expected that perivascular drainage receives relevance for diseases in which one or both of these proteins are involved. Two of such diseases are AD and cerebral SVD (i.e., a degenerative disorder of small arteries and arterioles). Aβ is the major protein component of senile plaques in AD[27] and the APOE ε4 allele is the major risk factor for sporadic AD[28]. In SVD, apoE is found in vessel wall lesions[21] and its ε4 allele is associated with the severity of the disease[29].

Alterations of the perivascular drainage of Aβ are found in AD as indicated by the findings that (1) Aβ is cleared through the perivascular space[1] in addition to its cellular uptake and degradation[4,6,8,30], and its clearance across the capillary BBB segment[25,31,32]; (2) cerebral amyloid angiopathy (i.e., the deposition of Aβ in the vessel wall near the basement membrane[33] and the perivascular space) occurs, strongly suggesting that Aβ clearance along the basement membranes and the perivascular spaces is insufficient in AD cases and, thereby, leads to the deposition of Aβ in the vessel walls[11,12,13,20]; and (3) apoE colocalizes with Aβ in the perivascular space[12,21], indicating that the interaction between Aβ and apoE is relevant for perivascular drainage. Therefore, differences in the Aβ-apoE interactions due to the APOE genotype[34] may modify the efficiency of this drainage pathway for Aβ and subsequently lead to the development of AD-related changes in the brain. The astrocytes of the glia limitans, thereby, appear to play an important role for the clearance mechanism. In an animal model expressing human apoE in astrocytes, glia limitans astrocytes exhibited human apoE and secreted it into the perivascular space[12]. In the human AD brain, these perivascular apoE-expressing astrocytes also contained Aβ[21], whereas non-AD cases showed Aβ-negative perivascular astrocytes most frequently[21]. Thus, the glia limitans function seems to be altered in AD.

In SVD, apoE is found in lesions of the vessel wall[21]. These lesions do not only contain apoE, but also other plasma proteins, such as immunoglobulin G (IgG), indicating leakage of plasma proteins into the vessel wall and, in most cases with SVD, also into the perivascular space[21]. Thus, SVD, which is often associated with hypertension[35], leads to a leakage of plasma proteins into the perivascular space caused by a mechanical alteration of the BBB precapillary segment (Fig. 2). Additionally, hypertension leads to an increased pinocytosis in endothelial cells of the arteriolar segment[36] and to a leakage of plasma proteins into the brain[37], pointing to an increased plasma transport through endothelial cells in hypertension.

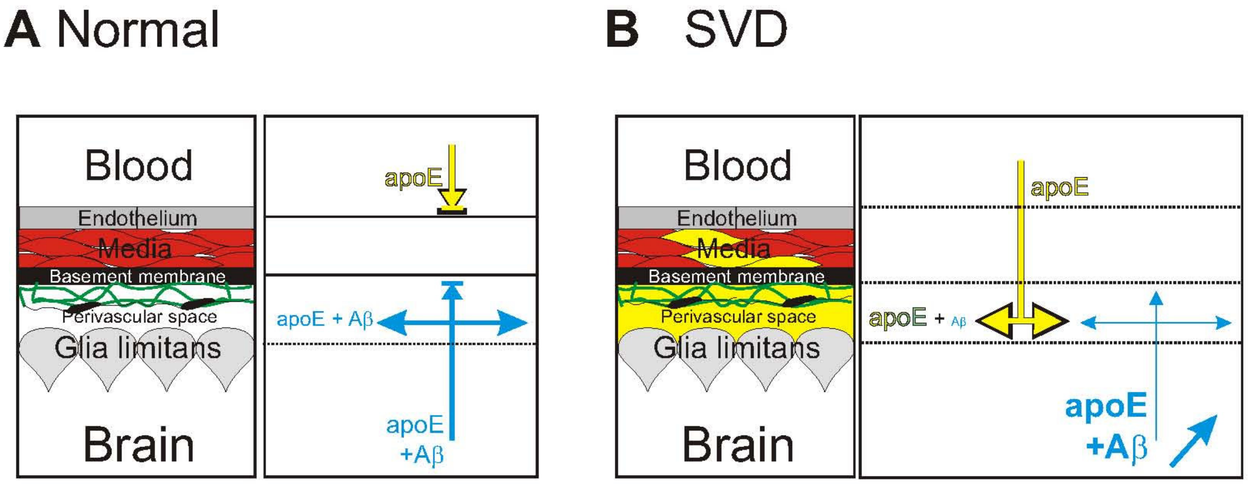

**FIGURE 2.** Schematic representation of the SVD-induced alteration of the precapillary segment of the BBB. (A) Physiological situation. (B) SVD-related BBB alteration. Due to endothelial lesions and the high arterial blood pressure, plasma leaks into the media of the arteries and finally into the perivascular space. Here, plasma proteins, including apoE, compete with the extracellular fluid, including brain-derived apoE and Aβ, for drainage. This competition leads to an insufficient clearance of brain apoE and Aβ, and finally to the accumulation of Aβ in the brain and the predominance of apoE-cleavage fragments in relation to full-length apoE[21].

Since SVD and its expansion throughout the brain, as well as the expansion of SVD-related MRI hyperintensities, are associated with the development of AD[38,39,40], the involvement of alterations in the perivascular drainage in both disorders may point to a pathogenetic link between both disorders. Hence, apoE may play an important role, since the clearance of apoE-bound Aβ from the brain parenchyma will be reduced in the event that plasma containing apoE leaks into the perivascular space and competes for the perivascular drainage (Fig. 2). Such a competitive inhibition of Aβ clearance into the perivascular space and into the CSF may explain the well-known decrease of Aβ in the CSF of AD patients[41,42].

## CONCLUSIONS

The pre- and postcapillary segments of the BBB are usually not significantly involved in the physiological exchange mechanisms between the brain parenchyma and the blood. However, the perivascular spaces and the vascular basement membranes allow the drainage of extracellular fluid and proteins from the brain into the CSF and the cervical lymph nodes[1,10]. The glia limitans, thereby, represents the border between the brain parenchyma and the perivascular fluid (Fig. 1). In the event that vessel disorders or inflammation cause a leakage of plasma into the perivascular space (e.g., SVD in the precapillary segment; encephalomyelitis disseminata in the postcapillary segment), the BBB is altered although the capillary segment may still be intact. Although there may be no direct influx of blood plasma into the brain parenchyma, the plasma with its proteins competes with the extracellular fluid for the perivascular drainage pathway that is usually reserved for the brain extracellular fluid (Fig. 2). In so doing, the drainage capacity for brain extracellular fluid and proteins could be reduced, leading (for example, in sporadic AD) to the retention of Aβ and apoE in the brain, triggering the cascade of AD-related neurodegeneration (Fig. 2).

Thus, alterations of the precapillary segment of the BBB seem to play an important role in the development of sporadic AD. SVD is, thereby, one important possible cause for precapillary BBB alterations.

## ACKNOWLEDGMENTS

Parts of the work, reviewed here, were supported by the DFG (grant Nos. TH624/4-1, TH624/4-2). The author thanks Dr. E. Ghebremedhin for critical reading of the manuscript.
